# Lung Function and Dementia Risk in the Atherosclerosis Risk in Communities (ARIC) Study

**DOI:** 10.1093/geroni/igab046.2465

**Published:** 2021-12-17

**Authors:** Srishti Shrestha, Xiaoqian Zhu, Stephanie London, Kevin Sullivan, Pamela Lutsey, B Gwen Windham, Michael Griswold, Thomas Mosley

**Affiliations:** 1 University of Mississippi Medical Center, Jackson, Mississippi, United States; 2 National Institute of Environmental Health Sciences, Research Triangle Park, North Carolina, United States; 3 The University of Mississippi Medical Center, Jackson, Mississippi, United States; 4 School of Public Health, University of Minnesota, Minneapolis, Minnesota, United States

## Abstract

Poor lung function has been linked with adverse neurocognitive outcomes including dementia, but evidence from well-designed prospective studies is limited. We therefore examined the association between lung function, as measured by forced expiratory volume in 1 second (FEV1) and forced vital capacity (FVC), and dementia risk in 12,688 participants of the ARIC study, a prospective study of adults aged 46-70 years (at index visit, mean age =57y, 45% male, 76% White) from four US communities. Lung function was assessed in 1991-1992 (index visit, 76% normal, 16% obstructive, and 8% restrictive lung function), and dementia was ascertained through 2019 via in-person assessments, telephone interviews, and medical record surveillance, with adjudication of dementia with all in-person exams. A total of 2452 developed dementia over 30 years of follow-up. We used Cox proportional hazards model to estimate hazard ratio (HR) and 95% confidence intervals (CI), adjusting for potential confounders (socio-demographics, behavioral factors, cardiovascular risk factors, APOE ε4). Higher FEV1 and FVC were associated with reduced dementia risk [(HR: 0.86, 95%CI: 0.78-0.98, per 1L increase in FEV1) and (HR: 0.86, 95%CI: 0.80-0.93 per 1L increase in FVC)]. Compared to normal lung function, restrictive disease was associated with elevated dementia risk [(HR: 1.19, 95%CI: 1.01-1.41), n=168 dementia cases]; HR for obstructive disease, though modestly elevated (1.09, 95%CI: 0.96-1.24, n=713 dementia cases), was not statistically significant. Our findings of decreased dementia risk with better lung function may have important implications in reducing burden of dementia that is attributable to environmental exposures and associated lung function impairment.

